# Unveiling autism spectrum disorder in South East Asia through a public health Lens

**DOI:** 10.3389/frcha.2024.1489269

**Published:** 2024-12-04

**Authors:** Alok Kumar, Sudip Bhattacharya

**Affiliations:** Department of Community and Family Medicine, All India Institute of Medical Sciences, Deoghar (AIIMS Deoghar), Deoghar, India

**Keywords:** autism, autism spectrum disorder, mental health, community prevention, developmental delay

## Abstract

Autism spectrum disorder (ASD) is a multifaceted developmental condition characterized by persistent challenges in social communication, restricted interests, and repetitive behaviors. Though there is no cure, early and intensive interventions can significantly improve the quality of life for those affected. The aim of this paper is to examine the complexities of autism spectrum disorder (ASD) from a public health perspective in South East Asian region, highlighting the global rise in prevalence and the compounded challenges posed by the COVID-19 pandemic. The rise in ASD prevalence from 4 to 5 cases per 10,000 children in the 1980s to 11.3 per 1,000 children in 2012 highlights the need for effective interventions. The pandemic exacerbated behavioral issues, anxiety, and screen time-related health problems, underscoring the importance of adjusting strategies for early identification and support. Diagnostic tools like the Modified Checklist for Autism in Toddlers (M-CHAT) and the Social Communication Questionnaire (SCQ) play a critical role in community-based screening. Effective prevention strategies include primary measures such as public awareness campaigns and genetic counseling, secondary measures focusing on early identification and intervention, and tertiary measures involving ongoing support and therapy. Addressing implementation challenges, particularly in low-income countries, requires enhanced public awareness, training of community health workers, and integration of ASD services into primary healthcare systems. Future research should aim to develop and evaluate scalable, culturally relevant interventions and explore the impact of environmental factors on ASD. Comprehensive strategies at the community level, combined with robust public health policies, are crucial for improving outcomes for individuals with ASD and their families.

## Introduction

Autism spectrum disorder (ASD) is a complex developmental condition characterized by persistent challenges in social communication, restricted interests, and repetitive behaviors, as outlined by the DSM-5 (Diagnostic and Statistical Manual of Mental Disorders, Fifth Edition) (1). Although autism is considered a lifelong condition, the degree of impairment varies significantly among individuals (2). ASD encompasses what were once distinct disorders, including Asperger's syndrome, childhood disintegrative disorder, autism, and an unspecified type of pervasive developmental disorder. The term “spectrum” reflects the broad range of symptoms and their varying degrees of severity. For a diagnosis, these symptoms must be present from early developmental stages, although they may not become fully apparent until social demands exceed the individual's capacities (3). Additionally, these symptoms must cause clinically significant impairment in social, occupational, or other important areas of functioning and cannot be better explained by intellectual disability or global developmental delay (4). While there is no known cure for autism spectrum disorder, many children can significantly benefit from intensive, early intervention. The causes of autism spectrum disorder are not fully understood, likely due to the disorder's complexity and the wide variation in symptoms and severity. Both genetic and environmental factors are believed to contribute. Multiple genes appear to be involved in ASD (5). For example, ASD can be associated with genetic disorders such as Rett syndrome or fragile X syndrome. Some genetic mutations are inherited, while others occur spontaneously. Environmental factors, such as viral infections, medications, complications during pregnancy, or exposure to air pollutants, may also play a role in triggering ASD (6).

Several risk factors are linked to a higher likelihood of developing ASD, including having a sibling with ASD, older parental age, certain genetic conditions (such as Down syndrome or fragile X syndrome), and very low birth weight. Boys are about four times more likely than girls to develop ASD (7). The challenges associated with ASD, including difficulties with social interactions, communication, and behavior, can lead to significant problems in school, learning, employment, independent living, social relationships, family stress, and vulnerability to victimization and bullying (8). The aim of this paper is to examine the complexities of autism spectrum disorder (ASD) from a public health perspective, highlighting the global rise in prevalence and the compounded challenges posed by the COVID-19 pandemic.

## Global and national burden of ASD

**Figure 1:**
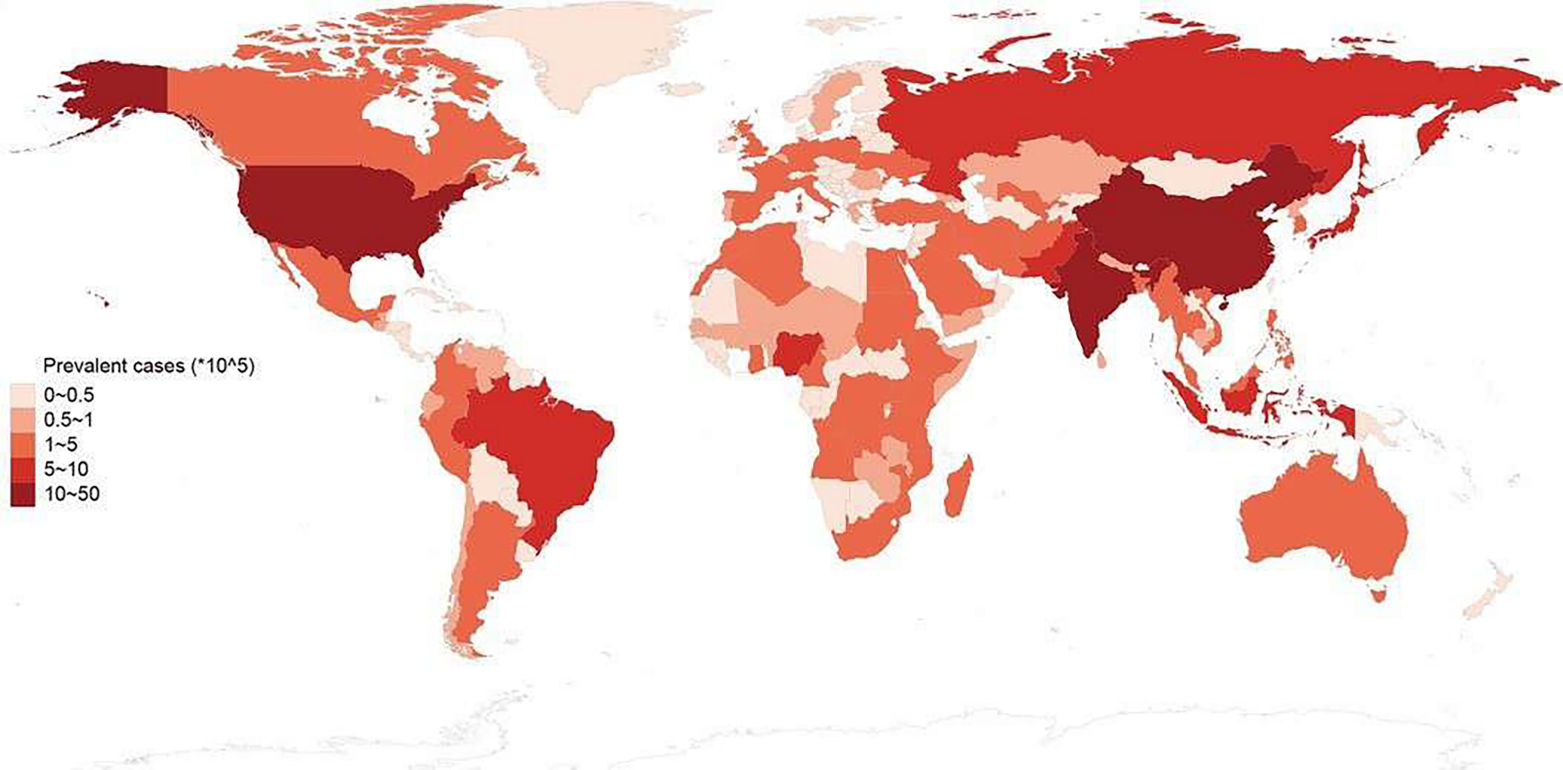
The estimated prevalence of autism spectrum disorder.

The prevalence of autism has increased significantly over the years. In the 1980s, the reported prevalence was approximately 4–5 cases per 10,000 children. By the 1990s, this number had risen to about 30–60 cases per 10,000 children (9). A 2020 study found that the combined prevalence of autism spectrum disorder (ASD) was 27.6 per 1,000 children aged 8 years, or roughly 1 in 36 children (10). Another study in Australia reported a prevalence of 1 per 255 children aged 2–17 years, with a tenfold increase over a 16-year period, consistent across all levels of child intellectual functioning (11). A Danish study, analyzing data from the Danish Psychiatric Central Research Registry, examined trends in the incidence of diagnosed ASD from 1995 to 2010. It identified 14,997 patients, with incidence rates increasing from 9.0 to 38.6 per 100,000 person-years over the 16-year period, particularly among females, adolescents, adults, and those diagnosed with Asperger's syndrome or PDD-NOS (12). In the United States, the estimated prevalence of ASD rose from 1.1% in 2008 to 2.3% in 2018, likely due to changes in DSM-5 diagnostic criteria, improved screening and diagnostic tools, and increased public awareness (13). Globally, in 2023, the pooled prevalence estimate for ASD was 0.72% (95% CI = 0.61–0.85) (14). In India, a community-based study on autism prevalence among children aged 1 to 10 years in Himachal Pradesh reported a prevalence of 15 per 10,000 (0.15%) (15). A 2017 study from Kolkata found the prevalence of ASD in school-going children to be 23 per 10,000 (16). The global impact (Figure 1) of ASD has significant effects on family dynamics, healthcare resources, and educational systems. Supporting an individual with ASD often entails a high lifetime cost, including medical expenses, educational interventions, and lost productivity (17).

## Changing pattern of ASD

The COVID-19 pandemic had a range of negative, neutral, and positive effects on both children with autism spectrum disorder (ASD) and their mothers, who worked to overcome the challenges presented by the crisis (18). Research indicated a significant increase in behavioral issues among children and adolescents with ASD, with 521 out of 1,004 individuals (51.9%) exhibiting behavioral changes such as conduct problems, emotional difficulties, aggression, and hyperactivity. Additionally, studies reported heightened anxiety and challenges in emotion regulation among this group, though one study noted clinical stabilization in some children with ASD during the pandemic (19). A large majority of families and caregivers—82.7% (544 out of 658)—faced significant challenges during this period. Most parents observed declines in their children's sleep quality (69.4%), behavioral regulation (52.8%), and acquired skills across various domains (54.2%) (20). Parental stress and related demands also escalated during the pandemic compared to pre-pandemic levels (21). At the onset of the COVID-19 pandemic, the suspension of school activities caused sudden disruptions in the daily routines of children and teenagers. These changes, coupled with physical distancing from friends, confinement to their homes, and exposure to distressing information about the pandemic, heightened feelings of stress, anxiety, and sadness. The excessive use of screens during the pandemic had numerous adverse effects on children and adolescents, leading to a higher incidence of visual impairments, sedentary lifestyles, unhealthy eating habits, weight gain, impaired sleep quality, and negative impacts on mental health. While digital platforms offer valuable educational tools, they must be used judiciously to avoid these negative consequences. A significant decrease in the number of days of physical activity (from 4.17 to 2.27 days per week; *p* = 0.0006) and a notable increase in screen time during both weekdays (from 3.69 to 6.25 h; *p* = 0.007) and weekends (from 5.94 to 7.39 h; *p* = 0.004) were observed during the pandemic (22). A survey conducted in India found that about 54% of children had more screen time than usual during the lockdown (23). The pandemic's impact on autistic children was further exacerbated by limited access to essential services and financial difficulties due to service interruptions, even after services resumed (24).

Children with more than two hours of daily screen exposure were found to have more speech delays and communication difficulties compared to those with less than two hours of exposure (25). Another study revealed that prolonged screen exposure of more than three hours per day was associated with an increased prevalence of ASD symptoms (26). It was also found that the primary reason for increased screen exposure was busy parents seeking to keep their children entertained. However, after reducing screen time, there was a significant improvement in the children's symptoms (27).

## Diagnosing ASD at community level

Various tools are available for diagnosing ASD at the community level. The Modified Checklist for Autism in Toddlers (M-CHAT) is a two-step parent questionnaire that takes about 10 min to complete and consists of 20 yes/no questions designed to screen children aged 16–30 months who are at high risk for ASD (28). The Social Communication Questionnaire (SCQ), available in many Indian languages, is often considered the gold-standard questionnaire used in autism research studies (29). Existing tools, such as M-CHAT and SCQ, while effective in Western contexts, may not adequately capture the unique cultural and social nuances of non-Western societies. Developing diagnostic tools that reflect local behaviors, traditions, and community dynamics is essential for accurate identification and assessment of ASD. That's why culture specific few tools have been adopted in Indian context (LMICs). As an example, the Trivandrum Autism Behavioral Checklist (TABC), developed and validated in Indian context, evaluates four major domains: social interaction, communication, behavioral traits, and sensory integration (30). Diagnosing ASD involves using established criteria, such as those in the Diagnostic and Statistical Manual of Mental Disorders, Fifth Edition (DSM-5), along with specific diagnostic tools like the Autism Diagnostic Observation Schedule (ADOS) and the Indian Scale for the Assessment of Autism (ISAA) (31). Other tools include the Knowledge about Childhood Autism among Health Workers (KCAHW) and the Opinion on the Availability of Facilities and Laws for Caring for the Needs and Rights of Children with Autism and Other Developmental Disorders (OFLCA) questionnaires (32).

At a micro level, educating family members to recognize ASD symptoms and bring the child in for screening can be crucial. Studies suggest that parental interventions can improve parenting confidence and, to a lesser degree, mental health (33). At the meso level, community healthcare workers, such as Accredited Social Health Activists (ASHA), Anganwadi Workers (AWW), and Auxiliary Nurse Midwives (ANM), can be instrumental in diagnosing ASD. For instance, the COMPASS trial, which includes the PASS Plus intervention alongside treatment as usual, involves ASHAs as one of the agents delivering the intervention (34). Studies also support the effectiveness of non-specialist health workers in delivering autism treatment in Asia (35).

At a macro level, policy interventions are necessary. For example, the Rashtriya Bal Suraksha Karyakram (RBSK) was launched in 2013 for community-level screening, early identification, and management of chronic conditions, including birth defects, diseases, deficiencies, developmental delays, and disabilities, from birth to 18 years (36). Healthcare is provided in district hospitals with Special Newborn Care Units and District Early Intervention Centers (DEIC). Infants are screened at delivery points or at home under the Home-Based Newborn Care package. Pre-schoolers and school-aged children are evaluated by mobile health teams using standardized tools in anganwadi centers and schools, respectively, with referrals managed at higher centers. The Rights of Persons with Disabilities Act, 2016 (RPWD Act) covers 21 types of disabilities, including ASD (37).

## Prevention of ASD

The Early Start Denver Model (ESDM) is a behavioral therapy for young children with ASD that focuses on social communication and play skills (38). In the U.S., children receiving intensive ESDM therapy showed significant improvements in IQ and adaptive behaviors compared to those receiving standard interventions (38). However, the therapy's annual cost of $40,000–$60,000 makes it largely inaccessible in low-income countries with insufficient trained professionals. Similarly, Applied Behavior Analysis (ABA) therapy has gained traction in Saudi Arabia, with government support enhancing its availability (39). Yet, access remains limited to financially secure families, especially in urban areas. In the UK, the TEACCH approach has been integrated into public schools, demonstrating improvements in communication and behavioral skills for children with ASD (40). However, scaling such initiatives in developing countries faces challenges, including limited resources and a lack of trained staff.

The Community Health Worker model in India shows promise for early screening of developmental delays, but barriers persist due to inadequate training and urban-centric services (41). While medications like risperidone and aripiprazole help manage symptoms, their use is less common in developing countries due to cost and limited healthcare access (42). In contrast, developed nations integrate autism services into schools, facilitating early identification and support, while developing countries struggle with stigma, inadequate resources, and a lack of specialized teachers, leading to exclusion from mainstream education (43). Overall, while effective therapies exist, significant barriers remain in accessing these services across different countries, necessitating a multifaceted approach to improve accessibility and integration for families affected by ASD.

The approaches to autism prevention outlined here underscore the importance of early intervention, targeted strategies, and behavioral therapy. Although there is no known cure for autism, these methods can help individuals with autism spectrum disorder (ASD) lead fulfilling lives by mitigating symptoms, enhancing skills, and improving overall well-being. Primary prevention aims to reduce the incidence of autism by targeting broad populations and focusing on promoting healthy development and addressing risk factors associated with autism. This includes public awareness campaigns, early childhood education programs, and parental support initiatives designed to limit children's screen time. Genetic counseling is also crucial for families with a history of ASD, as it provides insights into potential risks and guides family planning decisions. Secondary prevention targets specific at-risk groups to reduce the prevalence or severity of autism. This involves early identification and intervention for children who exhibit early signs of autism or have a family history of the disorder. Effective strategies include diagnostic evaluations, early intervention services, and genetic counseling. Research has shown that early diagnosis of ASD leads to significant improvements in cognitive, language, and social-emotional functioning in affected children (44). Tertiary prevention focuses on individuals who have already been diagnosed with autism, aiming to maintain functional adaptations, ensure well-being, and prevent relapses. This approach includes providing effective support, therapies, and services throughout an individual's lifespan. Non-pharmacological therapeutic interventions, such as specialized diets, supplements, antioxidants, hormones, vitamins, complementary and alternative medicine (CAM) therapies, herbal remedies, camel milk, and cannabidiol, are used to manage ASD symptoms (45). Family support is also essential, offering resources and assistance to help families manage the emotional and financial challenges of caring for a child with ASD.

To comprehensively understand autism spectrum disorder (ASD), it is crucial to address the challenges faced by individuals as they transition from childhood into adolescence and adulthood. While early intervention is vital, the evolving needs of adolescents and adults, particularly concerning employment, independent living, and social integration, must also be recognized (46). The transition to adolescence presents unique challenges, as social interactions and adaptive behaviors can become more pronounced due to shifting societal expectations (47). Unfortunately, many countries offer inadequate services for adolescents with ASD, focusing primarily on early interventions like ABA or ESDM. This leaves a significant gap in support during a critical developmental phase.

In the U.S. and parts of Europe, specialized programs have begun to address these needs, with interventions like Cognitive Behavioral Therapy (CBT) showing promise in reducing anxiety and improving social functioning among adolescents (48). However, in developing countries, services targeting this demographic are scarce, leading to what is known as a service cliff, where adolescents lose access to essential therapies. As individuals transition into adulthood, challenges persist, including high unemployment rates, with many adults with ASD struggling to secure meaningful employment due to a lack of tailored services.

While vocational training programs in countries like the U.S. and UK help individuals develop job skills, such programs are often absent in low- and middle-income countries (49). Individuals with ASD frequently rely on family support, which can become unsustainable over time, especially as caregivers age. Supported living arrangements are crucial but rarely available in developing countries, further exacerbating the difficulties faced by adults with autism.

The need for vocational training becomes critical as adolescents prepare to enter adulthood, yet such opportunities remain largely inaccessible in many regions. Expanding services that focus on social skills, emotional regulation, and vocational readiness is essential for bridging the gap between childhood interventions and adult support. Governments should invest in programs that prioritize vocational training, while partnerships with private entities and NGOs can create more opportunities for individuals with ASD. Ultimately, a lifelong perspective on autism services is needed, encompassing early intervention, adolescent support, vocational training, and adult living assistance to enhance the quality of life for individuals with ASD and enable them to lead fulfilling, independent lives. The WHO's Mental Health Action Plan 2013–2030 and Resolution WHA73.10 urge countries to close gaps in early diagnosis, care, and rehabilitation for mental and neurodevelopmental conditions, including autism. They also call for addressing the social, economic, and educational needs of affected individuals and families, while enhancing surveillance and research.

Autism prevention strategies should be implemented using a life-course approach, considering the different stages of an individual's life from the prenatal period to old age. The focus of prevention efforts may vary depending on the age of the targeted population.

## Implementation challenges and possible solutions at community level

In low-income countries, many essential elements for organizing mental health services are lacking, including specialist mental health professionals to support the services and a reliable supply of medications (50). Government programs, such as those focused on maternal and child health, could allocate resources to include autism screening and interventions as part of broader developmental care initiatives. Similarly, integrating autism services with existing primary care systems would make these services more accessible and cost-effective. For instance, routine screenings for autism could be added to early childhood health checkups at primary healthcare centers, ensuring that children are assessed alongside other developmental milestones. Additionally, national programs that focus on disability, mental health, or child welfare, such as India's Rashtriya Bal Swasthya Karyakram (RBSK), could incorporate autism services into their mandate, further streamlining the delivery of care (51). Collaborating with NGOs, private sector partners, and international organizations could also provide essential financial and technical support, helping to scale these interventions more effectively. By addressing funding and integration into national programs, the paper would provide a more comprehensive strategy for the widespread implementation of autism services in developing countries. The low baseline preparedness of health systems across various sites highlights the need for comprehensive interventions at the levels of health care organization, health facilities, and communities to ensure the sustainable delivery of quality mental health care integrated into primary care. A study revealed significant cross-country variation in the implementation of community-based services, with mental hospitals still being the dominant model of care in most countries (52). However, a few countries have adopted a balanced care model that includes both community services and mental hospitals. Engaging all staff in these initiatives remains a challenge, although some initial resistance has been successfully addressed (53).

To succeed in developing countries, autism services must be built on a comprehensive plan that addresses resource limitations, cultural factors, and existing healthcare infrastructure. Raising public awareness and reducing stigma through community education campaigns is crucial; involving local leaders, teachers, and health workers has proven effective, as seen in Bangladesh (54).

Training healthcare workers to recognize autism and deliver basic interventions is essential. Models like India's Accredited Social Health Activists (ASHAs) can be adapted to include autism-specific tools, while non-specialist workers in rural areas can be trained in parent-mediated interventions, reducing costs and ensuring consistent support (55).

Integrating autism services into primary healthcare is practical, as these centers are often the first contact point for families. Training professionals to use basic autism screening tools can lead to early diagnoses, exemplified by India's Rashtriya Bal Swasthya Karyakram (RBSK) (36). Schools should also train teachers to recognize autism and incorporate vocational training for adolescents with ASD.

Establishing referral networks and specialized autism centers is essential for advanced diagnostic and therapeutic services. Networks connecting community centers with regional autism hubs can bridge rural-urban gaps, while specialized centers can train healthcare workers and deliver therapies like Applied Behavior Analysis (ABA) (56).

Financial barriers are significant; governments should prioritize autism care in national budgets and offer subsidies or insurance coverage for services. Partnerships with NGOs and the private sector can also expand access to affordable services.

Logistical challenges like transportation hinder access, particularly in rural areas. Mobile health units and telehealth services can provide screening and therapy in remote locations (57). Continuous monitoring and evaluation are necessary to measure outcomes and ensure sustainability.

By focusing on awareness, capacity building, service integration, and addressing financial and logistical barriers, developing countries can create sustainable, community-based autism services through collaboration among governments, healthcare providers, educators, and NGOs.

Overcoming the implementation challenges of autism spectrum disorder (ASD) at the community level requires a multifaceted approach that addresses gaps in awareness, resources, and service delivery. First, increasing public awareness through education campaigns can help reduce stigma and promote early detection, encouraging families to seek support. Training and empowering community health workers, such as ASHAs, ANMs, and Anganwadi workers, is essential to improve early identification and intervention, especially in resource-limited settings. Additionally, integrating ASD services into existing healthcare frameworks, such as primary health centers and community health programs, can enhance accessibility and continuity of care. Collaborating with local governments, NGOs, and international organizations can help mobilize resources and provide the necessary training and infrastructure. Finally, engaging families and caregivers as active participants in the care process can foster a supportive environment and ensure that interventions are culturally appropriate and sustainable.

Stigma surrounding developmental disorders significantly impedes the early identification, diagnosis, and treatment of individuals with autism spectrum disorder (ASD), especially in low- and middle-income countries (58). Traditional beliefs and misconceptions often lead families to avoid seeking help, fearing social ostracism or being labeled as “cursed.” This stigma not only affects individuals with ASD but also places an emotional burden on their families, who may refrain from pursuing interventions due to concerns about community judgment (59).

Cultural perceptions further influence how autism services are delivered. In many African and South Asian societies, disabilities are often associated with supernatural beliefs, causing families to seek traditional healers instead of medical professionals (60). For instance, reliance on faith healers in countries like Nigeria and India can delay access to essential early intervention services (59, 61). Moreover, cultural norms regarding gender roles can hinder access to care; in some regions, women, as primary caregivers, may lack the authority to seek help for their children without male consent, perpetuating isolation for children with ASD (62).

Culturally sensitive approaches to service delivery are vital, as interventions effective in Western contexts may not translate easily into non-Western settings without considering local beliefs and practices. For example, therapies like Applied Behavior Analysis (ABA) may require adaptation to align with local parenting styles (63). Research from countries like China indicates that societal pressures around academic achievement can skew interventions, prioritizing academic skills over critical areas like communication and emotional regulation (64).

Stigma also impacts public policy and resource allocation, as autism often remains a low priority within health and education systems in developing countries (65). This results in limited investment in ASD services, leading to gaps in access to trained professionals and specialized programs. To address these challenges, culturally relevant interventions should encompass community outreach to reduce stigma, public education campaigns, and collaborations with local leaders to shift narratives around developmental disabilities. For example, initiatives in Bangladesh have successfully engaged local religious leaders to promote autism awareness, improving access to services (66).

## Conclusion

Understanding Autism Spectrum Disorder (ASD) from a public health perspective requires acknowledging the complexities of its diagnosis, the impact of modern challenges such as increased screen time, and the need for a comprehensive approach to prevention and intervention. Both globally and in India, addressing the burden of ASD demands heightened awareness, enhanced diagnostic and support services, and targeted prevention strategies aimed at improving outcomes for individuals and families affected by the disorder.

## Way forward

Future research on autism spectrum disorder (ASD) at the community level should focus on developing and evaluating scalable, culturally relevant interventions that can be integrated into existing primary healthcare systems. For instance, studies could investigate the role of community health workers, such as Accredited Social Health Activists (ASHA) in India, in the early identification and support of individuals with ASD, particularly in underserved areas. Research should also assess the long-term outcomes of early intervention programs like the Early Start Denver Model or Parent-Implemented Interventions and their adaptability in different cultural contexts (67). Additionally, exploring the impact of environmental factors, such as dietary influences or increased screen time, on the prevalence and severity of ASD in various communities could provide important insights. Investigating strategies to enhance community awareness and reduce stigma, similar to public awareness campaigns about mental health, will be crucial in ensuring timely and appropriate care for affected individuals.

## Data Availability

The original contributions presented in the study are included in the article/Supplementary Material, further inquiries can be directed to the corresponding authors.
